# Relationship between human herpesvirus 6 infection and inflammatory bowel disease using novel biomarker

**DOI:** 10.1002/jgh3.12992

**Published:** 2023-11-02

**Authors:** Satohiro Matsumoto, Yuzo Otaki, Yukio Yoshida, Nobuyuki Kobayashi, Naomi Oka, Hiroyuki Yanagisawa, Kazuhiro Kondo

**Affiliations:** ^1^ Department of Gastroenterology Jichi Medical University Saitama Medical Center Saitama Saitama Japan; ^2^ Department of Virology The Jikei University School of Medicine Minato City Tokyo Japan; ^3^ Department of Public Health & Environmental Medicine The Jikei University School of Medicine Minato City Tokyo Japan

**Keywords:** Crohn's disease, human herpesvirus 6, SITH‐1, ulcerative colitis

## Abstract

**Objective:**

Inflammatory bowel disease (IBD) is closely related to stress and fatigue. Human herpesvirus 6B (HHV‐6B) is reactivated by stress and fatigue and is associated with IBD. This study aimed to clarify the relationship between IBD and HHV‐6B.

**Methods:**

Antibody titers to SITH‐1, a protein specific to HHV‐6B latent infection, were measured in 163 patients with IBD (107 with ulcerative colitis [UC] and 56 with Crohn's disease [CD]); clinical and endoscopic scores and depression scores of UC and CD were analyzed to examine the relationship between SITH‐1 and IBD. The SITH‐1 cut‐off value was set as 1.96, according to known reports.

**Results:**

In patients with UC, C‐reactive protein (CRP) level was significantly higher (1.5 *vs* 0.6 mg/L, *P* = 0.006) and disease exacerbation within 6 months after entry was significantly more common in the SITH‐1 (+) group (20% *vs* 0%, *P* < 0.001). In the subanalysis comparing with and without UC exacerbation, the optimal cut‐off value for SITH‐1 to detect UC exacerbation was 3.44 (area under the curve: 0.81; 95% confidence interval: 0.72–0.90). CRP levels, SITH‐1 levels, and disease activity scores by the clinical or endoscopic index were significantly higher in the exacerbation group than in the non‐exacerbation group (2.6 *vs* 0.9 mg/L, *P* = 0.03; 4.90 *vs* 1.71, *P* < 0.001; 4 *vs* 3, *P* = 0.03; 5 *vs* 3, *P* = 0.02; respectively).

**Conclusion:**

Patients with UC with high titers of SITH‐1 have high disease activity and frequent disease exacerbation. SITH‐1 can be associated with UC disease activity.

## Graphical abstract

In patients with ulcerative colitis, C‐reactive protein level was significantly higher (1.5 *vs* 0.6 mg/L, *P* = 0.003) and disease exacerbation within 6 months after entry was significantly more common in the SITH‐1 (+) group (20% *vs* 0%, *P* < 0.001). SITH‐1 levels were significantly higher in the exacerbation group than in the non‐exacerbation group (4.90 *vs* 1.71, *P* < 0.001).

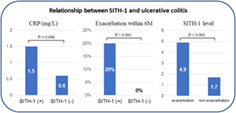

## Introduction

Inflammatory bowel disease (IBD) is a collective term for ulcerative colitis (UC) and Crohn's disease (CD), and it is a chronic intractable disease of unknown cause with periods of remission and relapse. IBD has been reported to occur more frequently in Western countries and to be rare in Asian and African countries, but the number of patients with IBD has been increasing worldwide in recent years. In Japan, the number of patients with both UC and CD has increased by approximately 10 times over the past 25 years, and the number of patients registered in the national epidemiological survey by the Health and Labor Sciences Research Group in 2016 has been reported to reach 220 000 for UC and 70 000 for CD.

Human herpesvirus 6 (HHV‐6) is reactivated by stress and fatigue and has been associated with IBD.^1^ In a study using samples of large‐intestinal mucosa from patients with IBD, the DNA detection rate of HHV‐6A/B was 9.1%, which was the third highest positive rate following 21.2% of Epstein–Barr virus (EBV) and 15.2% of cytomegalovirus (CMV).^2^ Kobayashi *et al*. discovered that SITH‐1, a protein specific to HHV‐6B latent infection, is expressed in the brain when HHV‐6B becomes reactivated from the latent state by stimuli such as stress. Furthermore, they developed a method to evaluate the expression of SITH‐1 by measuring anti‐SITH‐1 antibody titers in serum by ELISA.^3^ If the relationship between IBD and HHV‐6B can be determined using anti‐SITH‐1 antibody titers, SITH‐1 can not only become a new biomarker involved in the pathogenesis and progression of IBD, but also may pave the way for new methods of treating IBD with antiviral agents. This study aimed to determine the relationship between IBD and HHV‐6B using anti‐SITH‐1 antibody titers.

## Materials and methods

### Patients

This study was conducted prospectively as a joint research project of our center and the Department of Virology, the Jikei University School of Medicine. Of 454 patients with IBD (330 with UC and 124 with CD) who were regularly visiting and followed up at the Jichi Medical University Saitama Medical Center between January and June 2014, 168 patients aged ≥16 years who consented to participate in the study (109 with UC and 59 with CD) were enrolled in the study. After excluding 5 patients who could not undergo blood tests (2 with UC and 3 with CD), 163 patients (107 with UC and 56 with CD) were ultimately included (Table 1). All patients were divided into the SITH‐1 positive and negative groups and followed up. Background factors, contents of treatment, and clinical course were evaluated and analyzed for the entire cohort and for the UC and CD groups. Then, we divided subjects into two groups as follows: the exacerbation group, which required additional treatment (e.g. prednisolone, cytapheresis, biologics, among others) due to disease exacerbation within 6 months from entry, and the non‐exacerbation group, which required no additional treatment. We analyzed and evaluated the background factors, treatment contents, and clinical courses of both groups.

**Table 1:** Baseline characteristics

	All (*n* = 163)	UC (*n* = 107)	CD (*n* = 56)
Age, years	42 (32–55)	44 (37–58)	36 (25–48)
Gender: Male	93 (57%)	54 (50%)	39 (70%)
BMI	21.5 (19.5–24.2)	22.1 (19.9–24.2)	21.0 (18.9–22.8)
Smoking, current/ex/never	8/51/104	5/37/65	3/14/39
Age at diagnosis of IBD	29 (21–41)	32 (22–43)	24 (18–37)
Disease duration, years	10.0 (5.0–17.0)	10.0 (5.0–18.5)	8.8 (4.0–15.3)
Beck Depression Inventory	5 (1–11)	5.5 (1–10.8)	4.0 (1–11)
Depression's severity			
Normal	116 (71%)	76 (71%)	40 (71%)
Mild	33 (20%)	23 (21%)	10 (18%)
Moderate	12 (7%)	7 (7%)	5 (9%)
Severe	2 (1%)	1 (1%)	1 (2%)
Concomitant therapy			
Mesalazine	156 (96%)	101 (94%)	55 (98%)
Corticosteroid	18 (11%)	2 (2%)	16 (29%)
Azathioprine	56 (34%)	35 (33%)	21 (38%)
Anti‐TNF‐α	50 (31%)	13 (12%)	37 (66%)
History of surgery	16 (10%)	0	16 (29%)
History of depression	23 (14%)	20 (19%)	3 (5%)
Psychiatric outpatient	18 (11%)	13 (12%)	5 (9%)
Labo data			
Leukocyte count (10^9^/L)	5.5 (4.5–7.1)	5.5 (4.5–7.1)	5.6 (4.6–7.1)
Hemoglobin (g/dL)	13.6 (12.4–14.8)	13.7 (12.5–14.6)	13.6 (11.7–14.9)
Platelet count (10^9^/L)	259 (220–314)	256 (215–314)	261 (226–309)
Albumin (g/dL)	4.3 (4.1–4.6)	4.4 (4.1–4.6)	4.3 (3.9–4.6)
C‐reactive protein (mg/L)	0.9 (0.4–2.6)	0.9 (0.4–2.4)	1.2 (0.4–3.4)
SITH‐1 level	2.11 (0–4.98)	2.11 (0–4.98)	2.13 (0.03–4.98)
SITH‐1 (+)	84 (52%)	56 (52%)	28 (50%)

The data were presented as the median (interquartile range) or number (%).

BMI, body mass index; IBD, inflammatory bowel disease; TNF, tumor necrosis factor.

### Measurement of anti‐SITH‐1 antibody titers

Antibody titers to SITH‐1 were measured by indirect fluorescent antibody technique and ELISA in the serum of the target patients. In a study by Kobayashi *et al*., when the cut‐off value was set as 1.96 for the receiver operating characteristic (ROC) curve between anti‐SITH‐1 antibody titers and depression, the area under the curve (AUC) was 0.86, the antibody‐positive rates were 79.8% in patients with depression and 24.4% in healthy subjects, and the odds ratio (OR) was 12.2.^4^ Therefore, in this study, anti‐SITH‐1 antibody titers of 1.96 or higher and of less than 1.96 were defined as positive and negative, respectively.

### Sample collection

The sample required to measure antibody titers to SITH‐1 is approximately 1 mL of serum. Serum was obtained by collecting about 2 mL of blood into a collection tube for serum and centrifuging it. The extra amount of blood collected for the study was approximately 2 mL. The blood sampling was performed concomitantly with the blood testing performed in routine medical care. Collected blood samples were transferred for analysis to the Department of Virology, the Jikei University School of Medicine.

### Method of informed consent

The objective and method of this study were explained sufficiently to the target patients and healthy individuals using an informed consent form, and the study was conducted after obtaining written consent from both the patient and his or her legally authorized representative if the patient was between 16 and 20 years of age. If the symptoms were severe and the patient was incapable of giving consent, samples were first collected with the consent of the patient's legally authorized representative. In the case where the subject later became able to make a decision, the subject's intention was respected, and the samples were destroyed if consent for the study could not be obtained. This study was approved by the Ethical Review Board of Jichi Medical University Saitama Medical Center (S13‐23) and was conducted in compliance with the Declaration of Helsinki and ethical guidelines for clinical studies.

### Assessment of clinical and endoscopic disease activity

Clinical symptoms of UC were scored using the clinical activity index (CAI) established by Lichtiger *et al*.^4^: CAI ≤3 was defined as clinical remission, 4–6 as mild disease, 7–10 as moderate disease, and CAI ≥11 as severe disease. Endoscopic findings were quantified using the endoscopic index (EI) described by Rachmilewitz.^5^ The scores for each patient were determined by assessing areas with the most severe inflammation. Endoscopic remission was defined as an EI score of 4 or less. Exacerbation of UC was defined as relapse requiring additional treatment (e.g. prednisolone, cytapheresis, biologics, among others).

Clinical symptoms of CD were scored using the Harvey–Bradshaw index (HBI)^6^: HBI ≤4 was defined as clinical remission, 5–7 as mild disease, 8–16 as moderate disease, and HBI ≥17 as severe disease. Endoscopic findings were quantified using the Simple Endoscopic Score for Crohn's Disease (SES‐CD).^7^ Endoscopic remission was defined as an SES‐CD score of 2 or less. Exacerbation of CD was defined as relapse requiring a dose escalation of biologics, switching to other biologics, and the addition of prednisolone or thiopurines.

### Evaluation of severity of depression

The self‐scored Beck Depression Inventory questionnaire was used to evaluate the presence or absence of depressive symptoms.^8^ Questions consist of 21 items that are scored on a scale of 4 points, 0–3 per item, with a score range of 0–63 points. The score of 0–9 was defined as normal range, 10–18 as mild to moderate depression, 19–29 as moderate to severe depression, and 30–63 as severe depression.

### Statistical analyses

The data were presented as the median (interquartile range), and the Wilcoxon test was utilized for univariate analysis of background factors. For clinical biomarkers and disease exacerbation, we determined the cut‐off value for each score using the Youden index from the ROC curve and calculated the sensitivity, specificity, and accuracy. Variables with *P* values less than 0.05 in the univariate analysis were entered into a multivariate logistic regression analysis model to identify significant factors. All statistical analyses were performed using EZR (The R Foundation for Statistical Computing, version 1.54). Differences were regarded as significant at *P* values less than 0.05.

## Results

Table 2 shows the comparison between the SITH‐1 (+) and SITH‐1 (−) groups of patients with UC. There were no significant differences in sex, age, therapeutic products, the Beck Depression Inventory score, and depression severity. C‐reactive protein (CRP) level was significantly higher in the SITH‐1 (+) group (1.5 *vs* 0.6 mg/L, *P* = 0.006). Hemoglobin levels were significantly higher in the SITH‐1 (+) group (*P* = 0.04). Exacerbation of UC within 6 months after entry was significantly more common in the SITH‐1 (+) group (20% *vs* 0%, *P* < 0.001).

**Table 2:** Baseline characteristics in ulcerative colitis patients

	SITH‐1 (+) (*n* = 56)	SITH‐1 (−) (*n* = 51)	*P* value
Age, years	44 (38–59)	43 (37–56)	0.72
Gender: Male	31 (55%)	23 (45%)	0.34
BMI	23.0 (20.1–25.4)	21.2 (19.7–22.8)	0.05
Smoking, current/ex/never	4/22/30	1/15/35	0.20
Age at diagnosis, years	34 (22–45)	30 (22–42)	0.55
Disease duration, years	10.5 (5–17.8)	10.0 (6.0–18.5)	0.72
Extent at diagnosis, total colitis/left‐sided colitis/proctitis	42/10/4	37/11/3	0.94
Disease activity			0.21
Remission	37 (66%)	38 (75%)	
Mild	16 (29%)	7 (14%)	
Moderate	2 (4%)	4 (8%)	
Severe	1 (2%)	2 (4%)	
CAI	3 (2–4)	3 (2–3.5)	0.71
Endoscopic activity			0.83
Remission	32 (57%)	28 (55%)	
Non‐remission	18 (32%)	14 (27%)	
EI	3 (0–5)	3 (0–5)	0.73
Beck Depression Inventory	4 (1–8.5)	7 (1.5–12)	0.13
Depression's severity			0.33
Normal	42 (75%)	33 (65%)	
Mild	11 (20%)	12 (24%)	
Moderate	2 (4%)	5 (10%)	
Severe	0	1 (2%)	
Concomitant therapy			
Mesalazine	52 (93%)	49 (96%)	0.68
Corticosteroid	1 (2%)	1 (2%)	1
Azathioprine	18 (32%)	17 (33%)	1
Anti‐TNF‐α	7 (13%)	6 (12%)	1
History of depression	11 (20%)	9 (18%)	0.81
Psychiatric outpatient	5 (9%)	8 (16%)	0.38
Labo data			
Leukocyte count (10^9^/L)	5.8 (4.7–7.5)	5.4 (4.1–6.5)	0.14
Hemoglobin (g/dL)	14.0 (12.7–14.8)	13.4 (12.4–14.2)	0.04
Platelet count (10^9^/L)	266 (220–313)	256 (209–316)	0.75
Albumin (g/dL)	4.4 (4.2–4.6)	4.3 (4.1–4.5)	0.43
C‐reactive protein (mg/L)	1.5 (0.6–4.8)	0.6 (0.3–1.5)	0.006
Exacerbation within 6 months	11 (20%)	0	<0.001

The data were presented as the median (interquartile range) or number (%).

BMI, body mass index; CAI, colitis activity index; EI, endoscopic index; TNF, tumor necrosis factor.

Table 3 shows the comparison between the SITH‐1 (+) and SITH‐1 (−) groups of patients with CD. The most common disease location was L3 (ileocolitis type), followed by L1 (ileal type) in the SITH‐1 (+) group, and L3 (ileocolitis type), followed by L2 (colitis type) in the SITH‐1 (−) group (*P* = 0.01). There were no significant differences in the Beck Depression Inventory score, depression severity, CRP level, and disease exacerbation within 6 months after entry between the SITH‐1 (+) and SITH‐1 (−) groups.

**Table 3:** Baseline characteristics in Crohn's disease patients

	SITH‐1 (+) (*n* = 28)	SITH‐1 (−) (*n* = 28)	*P* value
Age, years	36 (25–51)	36 (24–48)	0.90
Gender: Male, (%)	18 (64%)	21 (75%)	0.56
BMI	19.9 (18.8–22.8)	21.5 (19.8–22.8)	0.25
Smoking, current/ex/never	3/7/18	0/7/21	0.33
Age at diagnosis, years	24 (18–35)	24 (17–37)	0.82
Disease duration, years	6.5 (2.9–14.3)	12.5 (7.8–17.0)	0.11
Disease location, L1/L2/L3	10/4/14	2/11/15	0.01
Disease activity			0.15
Remission	21 (75%)	17 (61%)	
Mild	6 (21%)	5 (18%)	
Moderate	1 (4%)	6 (21%)	
Harvey–Bradshaw index	1 (0–4.3)	2.5 (0.8–7)	0.18
Endoscopic activity			0.77
Remission	12 (43%)	12 (43%)	
Non‐remission	10 (36%)	13 (46%)	
Simple Endoscopic Score for Crohn's Disease	2 (0–5)	3 (2–9)	0.23
Beck Depression Inventory	2.5 (0.8–12.3)	5 (1–8.5)	0.89
Depression's severity			0.34
Normal	18 (64%)	21 (75%)	
Mild	6 (21%)	4 (14%)	
Moderate	4 (14%)	1 (4%)	
Severe	0	1 (4%)	
Concomitant therapy			
Mesalazine	27 (96%)	28 (100%)	1
Corticosteroid	8 (29%)	8 (29%)	1
Azathioprine	10 (36%)	11 (39%)	1
Anti‐TNF‐α	16 (57%)	21 (75%)	0.26
History of surgery	10 (36%)	6 (21%)	0.38
History of depression	1 (4%)	2 (7%)	1
Psychiatric outpatient	3 (11%)	2 (7%)	1
Labo data			
Leukocyte count (10^9^/L)	4.9 (4.5–6.0)	5.7 (5.1–8.0)	0.11
Hemoglobin (g/dL)	13.3 (10.9–14.5)	14.4 (12.9–15.0)	0.07
Platelet count (10^9^/L)	256 (218–343)	264 (232–300)	0.83
Albumin (g/dL)	4.3 (3.6–4.5)	4.3 (4.1–4.6)	0.29
C‐reactive protein (mg/L)	1.0 (0.4–3.5)	1.3 (0.4–2.7)	0.95
Exacerbation within 6 months	3 (11%)	1 (4%)	0.61

The data were presented as the median (interquartile range) or number (%).

BMI, body mass index; L1, ileum type; L2, colon type; L3, ileum and colon type; TNF, tumor necrosis factor.

**Figure 1:**
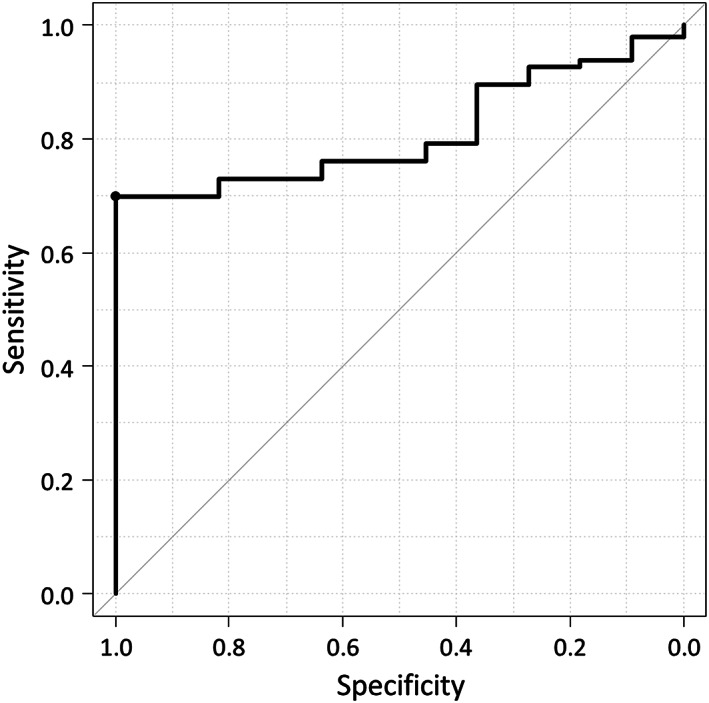
The receiver operating characteristic curve of SITH‐1 for predicting disease exacerbation.

Since a SITH‐1 cut‐off value of 1.96 only showed a significant difference in the exacerbation of UC, we analyzed the cut‐off value for SITH‐1 to detect UC exacerbation using the Youden index from the ROC curve. The AUC for SITH‐1 was 0.81 (95% confidence interval [CI]: 0.72 ***–*** 0.90), and the optimal cut‐off value for predicting disease exacerbation was determined to be 3.442; this value had a sensitivity of 0.67 and a specificity of 1 (Fig. 1). Based on this value, anti‐SITH‐1 antibody titers of 3.44 or higher and less than 3.44 were defined as positive and negative, respectively. Sensitivity, specificity, and accuracy were also calculated for the cut‐off value.

Eleven patients experienced UC exacerbation within 6 months from entry, three of whom were hospitalized. Table 4 shows the analysis results of the comparison between the exacerbation and the non‐exacerbation groups in UC patients. Mesalazine administration was significantly lower in the exacerbation group (73% *vs* 97%, *P* = 0.01). The rates of patients with clinical remission at study entry were significantly lower in the exacerbation group (36%) as compared to the non‐exacerbation group (74%) (*P* = 0.01). CRP levels were significantly higher in the exacerbation group than in the non‐exacerbation group (2.6 *vs* 0.9 mg/L, *P* = 0.03). Both clinical and endoscopic activity scores were significantly higher in the exacerbation group than in the non‐exacerbation group (*P* = 0.03; *P* = 0.02, respectively). In addition, SITH‐1 levels were significantly higher in the exacerbation group (4.90) than in the non‐exacerbation group (1.71) (*P* < 0.001).

**Table 4:** Clinical characteristics: comparison between the exacerbation and the non‐exacerbation groups in ulcerative colitis patients

	Exacerbation (*n* = 11)	Non‐exacerbation (*n* = 96)	*P* value
Age, years	40 (30–44)	46 (37–58)	0.18
Gender: Male	7 (64%)	47 (49%)	0.53
BMI	22.4 (19.2–23.2)	22.1 (19.9–24.3)	0.78
Smoking, current/ex/never	2/4/5	3/33/60	0.08
Age at diagnosis of IBD	31 (23–34)	32 (22–44)	0.42
Disease duration, years	11.0 (7–16.5)	10 (5–19.3)	0.88
Beck Depression Inventory	6 (1–8)	5 (1–11)	0.69
Disease activity			0.001
Remission	4 (36%)	71 (74%)	
Mild	5 (45%)	18 (19%)	
Moderate	1 (9%)	5 (5%)	
Severe	1 (9%)	2 (2%)	
Clinical remission, No	4 (36%)	71 (74%)	0.01
Depression's severity			1
Normal	9 (82%)	66 (69%)	
Mild	2 (18%)	21 (22%)	
Moderate	0	7 (7%)	
Severe	0	1 (1%)	
Concomitant therapy			
Mesalazine	8 (73%)	93 (97%)	0.01
Corticosteroid	1 (9%)	1 (1%)	0.20
Azathioprine	5 (45%)	30 (31%)	1
Anti‐TNF‐α	6 (55%)	11 (11%)	0.62
History of depression	3 (27%)	17 (18%)	0.43
Psychiatric outpatient	2 (18%)	11 (11%)	0.62
Labo data			
Leukocyte count (10^9^/L)	7.1 (4.9–8.5)	5.5 (4.5–6.9)	0.09
Hemoglobin (g/dL)	13.6 (11.9–14.9)	13.7 (12.5–14.5)	0.85
Platelet count (10^9^/L)	274 (220–295)	257 (220–313)	0.73
Albumin (g/dL)	4.4 (4.2–4.6)	4.3 (4.1–4.6)	0.67
C‐reactive protein (mg/L)	2.6 (0.9–3.0)	0.9 (0.4–2.0)	0.03
CAI	4 (3–5)	3 (2–4)	0.03
EI	5 (3–5)	3 (0–5)	0.02
SITH‐1 level	4.90 (4.60–10.6)	1.71 (0–4.20)	<0.001

The data were presented as the median (interquartile range) or number (%).

BMI, body mass index; CAI, colitis activity index; EI, endoscopic index; TNF, tumor necrosis factor.

The AUC for CRP was 0.69 (95% CI: 0.52–0.87) with a cut‐off value of 2.3 mg/L. In the multivariate logistic regression analysis model, significant variables for risk of UC exacerbation within 6 months included only achievement of clinical remission (OR 0.14, 95% CI: 0.02–0.82, *P* = 0.02) and not SITH‐1 cut‐off value of 3.44.

## Discussion

Herpesvirus can sustain a lifelong latent infection in a host, and it is an important component of the viral microbiome that sometimes increases the risk of various diseases.^9^, ^10^ HHV‐6 is classified into two species, HHV‐6A and HHV‐6B. Primary HHV‐6B infection occurs during childhood and presents as a sudden rash; subsequently, the virus is latent in almost 100% of humans. The primary HHV‐6A infection is unknown. During latency, HHV‐6B is most abundant in saliva and reaches the olfactory bulb, a part of the brain, from the nasal cavity, and the virus also becomes latent there. HHV‐6 in saliva produces the SITH‐1 protein based on the SITH‐1 gene and comprises 159 amino acids with a complex structure. Although SITH‐1 is expressed in the olfactory bulb in the brain, direct investigation of SITH‐1 expression is difficult. Therefore, Kondo *et al*. developed a method to detect the expression of SITH‐1 by serum antibody testing.^3^

Kobayashi *et al*. found that the OR of SITH‐1‐CAML complex antibody positivity affecting depression was as high as 12.2.^3^ Activation of the hypothalamic–pituitary–adrenal (HPA) axis is known to increase the risk of depression,^11^, ^12^ although there is a possibility that multiple factors may play a role in the effects of HHV‐6B and SITH‐1 on depression. Since immunosuppression due to excessively activated HPA axis and decreased immune function due to olfactory bulb dysfunction promote HHV‐6B proliferation in the brain, both HPA axis activation and olfactory bulb dysfunction may increase the risk of depression.^3^

Since anti‐SITH‐1 antibody titers are significantly higher in healthy individuals with depressive symptoms than in those without depressive symptoms, there is a possibility that SITH‐1 may indicate subclinical depression.^3^ This study found no significant association between SITH‐1 and depression. One of the reasons for this may be that the assessment using the Beck Depression Inventory questionnaire was a single survey conducted only at the time of study entry and that the course of depression and depressive symptoms were not followed up.

In this study, patients in the SITH‐1 (+) group with UC had significantly higher levels of CRP. Furthermore, they were more likely to experience exacerbation of IBD within 6 months after study entry. Previous research on UC found that compared to patients whose samples of large‐intestinal mucosa were negative for all EBV, CMV, and HHV‐6A/B, patients who were positive for at least two viruses were more likely to have undergone complete removal of the large intestine during a median observation period of 78.2 months (hazard ratio: 72.3; 95% CI: 1.85–2820).^2^ It is suggested that infection with or reactivation of these viruses may contribute to the worsening of intestinal inflammation. In this study, a comparison of the exacerbation and non‐exacerbation groups in UC patients showed that SITH‐1 levels were significantly higher in the exacerbation group, suggesting a relationship between SITH‐1 and disease activity. Since patients with UC exhibited high disease activity in the SITH‐1 (+) group, SITH‐1 may be a predictor of UC exacerbation. This may be related to the fact that the presence of depression or subclinical depression may affect UC exacerbation in SITH‐1‐positive patients. In order to demonstrate this, data on temporal changes in anti‐SITH‐1 antibody titers are needed.

In this study, we found differences between UC and CD in the association with SITH‐1. Furthermore, UC and CD are known to have different characteristics. For instance, appendectomy for appendicitis is associated with a reduced risk of developing UC,^13^ and dysbiosis is closely related to exacerbation of UC. Moreover, the efficacy of fecal microbiota transplantation for UC has been reported.^14^ Regarding the relationship between patients with IBD and HHV‐6, the prevalence of HHV‐6‐DNA was higher in UC tissue compared to CD tissue (76% *vs* 45%).^15^ In a study of 79 patients with IBD (47 UC and 32 CD) and 15 controls without IBD, HHV‐6B immunohistochemistry intensity correlated with endoscopic disease severity in UC but not CD.^1^ Although data on the association of patients with IBS with HHV‐6 are lacking, it is suggested that HHV‐6 is more closely related to UC than CD.

The study limitations include a single‐center study, a small sample size, a short observation period, and only a one‐time questionnaire survey on depression. Antibody titers to SITH‐1 were measured only once, and serological titers of other viruses, such as CMV, were not evaluated. Currently, fecal calprotectin and leucine‐rich alpha 2 glycoprotein are used in addition to CRP as surrogate markers for evaluating mucosal inflammation; however, these biomarkers had not yet emerged at the time this study was conducted. Finally, due to a single measurement of SITH‐1 and the possibility that SITH‐1 changes over time, the follow‐up period for disease exacerbation had to be as short as 6 months.

In conclusion, SITH‐1 (+) patients with UC have higher disease activity and many of them may subsequently experience relapse. This suggests that SITH‐1 can be associated with UC disease activity. Further larger scale, long‐term prospective studies are needed to confirm obtained results and validate the role of SITH‐1 titer in UC.
